# Preoperative *Staphylococcus aureus* nasal screening and decolonization for the prevention of periprosthetic joint infection in elective total joint arthroplasty: A single-center retrospective comparative study

**DOI:** 10.1051/sicotj/2026040

**Published:** 2026-10-23

**Authors:** Virginia Cinelli, Alessandro El Motassime, Giacomo Capece, Raffaele Vitiello, Giulio Maccauro, Luca Andriollo, Sébastien Lustig, Francesco Benazzo, Rudy Sangaletti

**Affiliations:** 1 Department of Orthopedics and Geriatric Sciences, Catholic University of the Sacred Heart Largo Francesco Vito, 8 00168 Rome Italy; 2 Department of Orthopedics, Ageing and Rheumatological Sciences, Fondazione Policlinico Universitario A. Gemelli IRCCS Largo Agostino Gemelli 8 00168 Rome Italy; 3 Ortopedia e Traumatologia, Fondazione Poliambulanza Istituto Ospedaliero Brescia Italy; 4 U.O.C. Orthopedics and Traumatology, Ospedale dei Pellegrini 80134 Naples Italy; 5 Artificial Intelligence Center, Alma Mater Europaea University 1090 Vienna Austria; 6 Univ Lyon, Claude Bernard Lyon 1 University, IFSTTAR, LBMC UMR_T9406 Lyon France; 7 Orthopaedics Surgery and Sports Medicine Department, FIFA Medical Center of Excellence, Croix-Rousse Hospital, Hospices Civils de Lyon, Lyon North University Hospital Lyon France; 8 IUSS Istituto Universitario Di Studi Superiori Pavia Italy

**Keywords:** Periprosthetic joint infections, PJI, Nasal swab screening, *Staphylococcus aureus*, Prevention

## Abstract

*Introduction: Staphylococcus aureus* is the most frequently implicated pathogen in periprosthetic joint infections (PJIs) and surgical site infections (SSIs), with nasal colonization being a well-established risk factor for their onset. Despite this association, the role of routine preoperative screening and decolonization in reducing postoperative infection rates remains debated. *Methods*: This retrospective study analyzed 2,507 elective joint arthroplasty procedures performed between January 2022 and December 2024 at a single center in Italy. Starting in 2024, patients underwent nasal swab screening for methicillin-sensitive *S. aureus* (MSSA) and methicillin-resistant *S. aureus* (MRSA). Positive carriers received a decolonization protocol consisting of mupirocin nasal ointment and chlorhexidine showers in the days preceding surgery. The incidence of PJIs in this group was compared to that observed in a control cohort from 2022–2023, during which no screening or decolonization was implemented. *Results:* Among the 988 patients screened in 2024, 231 (25.2%) tested positive for MSSA and 18 (1.8%) for MRSA. Notably, no PJIs caused by MSSA or MRSA were reported that year, compared to three such cases recorded in the preceding 2 years. Overall, 11 PJIs (0.44%) were documented across the study period. All infections in 2024 occurred in patients who had tested negative for *S. aureus* colonization. However, the reduction in infection rates did not reach statistical significance (*p* = 1.00). *Conclusions:* Although the observed decrease in *S. aureus*-related periprosthetic joint infections was not statistically significant, the trend suggests that preoperative screening and targeted decolonization may contribute to a lower incidence of these infections. These findings support further investigation into standardized preventive strategies to enhance outcomes in joint replacement surgery.

## Introduction

Notwithstanding its efficacy, total joint arthroplasty (TJA) is associated with risks and complications, among which periprosthetic joint infection (PJI) remains one of the most severe. PJI has an average incidence of approximately 1–2% [1–3], ranging between 0.5% and 2.4% in primary total hip arthroplasty (THA) and total knee arthroplasty (TKA), and increasing to 20% in revision procedures, accounting for 25% of TKA revisions [4]. Clinically, PJIs often require repeated surgical interventions and prolonged antibiotic treatments [5], and are associated with systemic complications [6], and increased healthcare costs [7].

The colonization of *Staphylococcus aureus* (*S. aureus*) is a well-documented risk factor for PJI and surgical site infections (SSI) in orthopedic procedures. Approximately 63% of PJIs are caused by *S. aureus* [8]. Studies indicate that approximately one-third of the general population carries *S. aureus*, primarily in the anterior nares, although colonization may also occur at extranasal sites [9, 10].

Hays et al. reported a significant rise in the incidence of methicillin-resistant *S. aureus* (MRSA)-related PJIs, particularly after 2004, with prevalence increasing from 0–5.8% to 13.6–19.2% [11]. MRSA infections are associated with extended hospital stays and increased postoperative mortality, with a mortality risk nearly twice that observed in methicillin-sensitive *S. aureus* (MSSA) infections [12]. These findings underscore the importance of assessing *S. aureus* colonization before surgery, as targeted interventions may reduce postoperative complications. Despite the evident clinical relevance of *S. aureus* screening and decolonization, no clear consensus has been reached regarding the routine implementation of such measures in the preoperative setting. The 2018 International Consensus Meeting (ICM) on Musculoskeletal Infection did not provide definitive recommendations on this issue due to insufficient supporting evidence [13]. Similarly, the European Bone and Joint Infection Society (EBJIS) and the Musculoskeletal Infection Society (MSIS) have not established standardized protocols [14, 15]. Existing studies indicate that individuals colonized with MRSA have a significantly higher risk of developing invasive infections compared with non-colonized patients [16]. The European Knee Associates (EKA) recommend MRSA nasal screening along with optimization of modifiable risk factors, including body mass index (BMI) <35, hemoglobin A1c <7.5, fructosamine <292 mmol/L, and smoking cessation in the preoperative period [17].

This retrospective study aimed to evaluate the prevalence of nasal carriers of MSSA and MRSA at a single Italian hospital and to assess the impact of a decolonization protocol, using mupirocin ointment and chlorhexidine showers, on reducing the incidence of PJIs. Additionally, infection rates were compared between patients who underwent screening and decolonization and a historical cohort that did not receive such intervention, in order to assess the effectiveness of this preventive strategy.

## Methods

### Study setting and design

A retrospective observational study was conducted following PROCESS guidelines, involving 2,507 patients who underwent TJA from January 2022 to December 2024. The study complies with national ethical standards and the Declaration of Helsinki. Written informed consent for data collection was obtained from all patients upon admission before their surgery, in accordance with institutional protocol.

### Inclusion and exclusion criteria

The inclusion criteria were as follows: (I) individuals aged 18 years or older, (II) absence of a prior history of joint infections, (III) no previous oncological history or existing pathology.

The exclusion criteria comprised: (I) follow-up duration of less than 6 months, (II) incomplete preoperative, intraoperative, or follow-up data, (III) a prior history of open fractures affecting the involved anatomical region.

### Patients

In a single center in Italy, between 2022 and 2024, a total of 2507 elective total joint prosthetic procedures were performed on patients who were found eligible according to inclusion and exclusion criteria. Table 1 presents the annual distribution of cases involving TKA, THA, and total shoulder arthroplasty (TSA) (Table 1).

**Table 1:** Synthetic presentation of TJA (total joint arthroplasty) [THA = total hip arthroplasty, TKA = total knee arthroplasty, TSA = total shoulder arthroplasty].

	THA	TKA	TSA	TJA
2022	301	395	46	742
2023	437	262	78	777
2024	557	382	49	988
Total	1295	1039	173	2507

### Perioperative procedures

Prior to hospital admission, patients undergo a comprehensive evaluation by the anesthesiology team. Only in 2024, as part of this assessment, a nasal swab is performed to screen for *S. aureus* colonization. In cases of a positive result, patients must follow the infection prevention protocol to reduce perioperative complications. Recommended pre-surgery decolonization measures include:

1. Intranasal decolonization: application of mupirocin nasal ointment 2% twice daily for 5 days to eliminate *S. aureus* in the nares [18, 19].
2. Preoperative antiseptic bathing: daily showers, including hair washing, with chlorhexidine digluconate for 5 days to lower skin microbial load.

On the day of the surgical procedure, skin disinfection is conducted using a 2% chlorhexidine digluconate solution, followed by the establishment of a sterile field using disposable towels. During surgical procedures, the use of diluted povidone-iodine (PID) irrigation is endorsed according to the guidelines established by the World Health Organization (WHO), the Centers for Disease Control and Prevention (CDC), and the International Consensus Meeting Clinical Practice Guidelines [20].

The institution’s standard perioperative antibiotic prophylaxis includes a preoperative dose of cefazolin (30 minutes before skin incision) and two postoperative doses at 12-hour intervals. For patients with a documented β-lactam allergy, we administer an intraoperative dose of vancomycin or teicoplanin, followed by an extra dose 12 hours post-surgery.

In patients who test positive for nasal swabs for MSSA and MRSA, prophylaxis consists of an intraoperative dose of vancomycin at a dosage of 15–20 mg/kg, in addition to the standard antibiotic regimen [17].

No drains or catheters were used in any case.

All patients were regularly monitored in the postoperative period. None were lost to follow-up for a minimum of 1 year.

A definitive diagnosis of PJI was confirmed when at least two intraoperative microbiological samples yielded the same organism, or when a single positive sample was obtained in conjunction with a sinus tract, elevated serum erythrocyte sedimentation rate or C-reactive protein, or an increased synovial leukocyte count or percentage of neutrophils, following the last guidelines. There were no instances of culture-negative PJI identified.

### Statistical analysis

Dedicated SPSS statistical calculation software (SOSS Inc., Chicago, IL, USA) was utilized. Data will be presented using means and standard deviations for quantitative variables, along with counts and percentages for qualitative variables. Results are reported with two decimal places, rounded appropriately. The Student’s *t*-test was employed for the statistical analysis of continuous variables, and the Fisher’s exact test was employed for the statistical analysis of discrete variables. A p-value of less than 0.05 was considered statistically significant.

## Results

In the three-year period from 2022 to 2024, there were 11 cases of PJI and SSI identified among 2,507 primary prostheses performed (0.44%).

Table 2 shows the yearly breakdown of infection cases categorized by pathogen type (Table 2).

**Table 2:** Distribution of periprosthetic joint infections (PJI) by year, joint location, and causative pathogen. Joint locations are reported as follows: THA = total hip arthroplasty, TKA = total knee arthroplasty, TSA = total shoulder arthroplasty [MSSA: methicillin-sensitive *S. aureus*; MRSA: methicillin-resistant *S. aureus*; Others: *Staphylococcus capitis*, *Staphylococcus epidermidis*, *Staphylococcus haemolyticus*, Corynebacterium, *Enterococcus avium*, *Enterococcus faecalis*, *Propionibacterium acnes*, *Streptococcus sanguinis*, and Moraxella spp.].

		THA	TKA	TSA	Total
2022	MRSA		1		1
	MSSA		1		1
	Others		2	1	3
2023	MRSA	1			1
	MSSA				
	Others	1	1	1	3
2024	MRSA				
	MSSA				
	Others		1	1	2
Total		2	6	3	11

In 2024, there were two cases of PJI by *S. epidermidis*.

A total of 988 swabs were performed, revealing that 231 tested positive for MSSA, which corresponds to 25.2% of the samples. Furthermore, 18 samples were positive for MRSA, making up 1.8% of the total.

The analysis showed that the patients who developed PJIs in 2024 had negative nasal swabs for both MRSA and MSSA.

A statistical analysis of the results showed that, when comparing all PJIs that occurred during the 2022–2023 biennium with those in 2024, during which nasal swab screening was implemented, the values were as follows: 9 vs 2, respectively (*p* = 0.2182).

A further analysis focusing exclusively on PJIs caused by MSSA/MRSA, compared to the total number of PJIs during the 2022–2023 biennium and contrasted with those in 2024, yielded the following results: 2 of 9 vs 0 of 2, respectively (*p* = 1.00).

## Discussion

The present study evaluated the impact of preoperative nasal screening and targeted decolonization for *S. aureus* on the incidence of periprosthetic joint infection in elective total joint arthroplasty. Although no statistically significant reduction was observed, no *S. aureus*-related infections occurred after implementation of the screening protocol in 2024. These findings suggest a potential protective effect of targeted decolonization within a structured perioperative prevention pathway. The comparison of MRSA and MSSA infection rates before and after the implementation of the decolonization protocol yields significant insights. In 2022, there was one case each of MRSA and MSSA among four infections. In 2023, only one case of MRSA was reported, with no MSSA infections, resulting in cumulative totals of two MRSA infections and one MSSA infection from eight documented PJIs during the pre-decolonization period. This reflects infection rates of approximately 25% for MRSA and 12.5% for MSSA. Following the introduction of the nasal screening and decolonization protocol in 2024, it is noteworthy that, despite higher percentages of positive nasal swabs for MSSA (25%) and MRSA (1.8%), no infections due to either pathogen were observed that year. This suggests the potential effectiveness of the decolonization protocol, even if the results are not statistically significant.

In this cohort, the prevalence of *S. aureus* carriers is consistent with previous descriptions in orthopedic populations, with MSSA carriers ranging from 20% to 30% (25.2%) and MRSA carriers between 2% and 4% (1.8%) [21]. In the Eastern United States, several studies highlighted higher rates, including 4.4% reported by Kim et al. [22], 5% by Baratz et al. [23], and 4.6% by Chen et al. [24]. Meanwhile, in the Western United States, a more modest rate of 1.8% was documented by Price et al. [25], with Moroski et al. [26] reporting a slightly elevated rate of 4.2%. Across the Atlantic, in the United Kingdom, Iqbal et al. [27] identified a rate of 1.8%, while Ahmad et al. [28] noted a slightly higher rate of 2.2%. Furthermore, in Spain, Valverde et al. discovered an MRSA positivity rate of 1.01%. These findings collectively illustrate the varying prevalence of MRSA across different regions, emphasizing the importance of ongoing surveillance in understanding its spread.

Nasal colonization is a modifiable risk factor for infection rates, leading to the establishment of screening protocols using nasal swabs and decolonization treatments in various medical settings. Mupirocin is the most commonly used agent for this purpose. Evidence indicates that this agent effectively diminishes the incidence of postoperative *S. aureus* infections among surgical patients who are carriers of *S. aureus* [29]. Kalmeijer et al. conducted a double-blind study comparing mupirocin to a placebo, revealing a significantly higher eradication rate among nasal carriers within the mupirocin group (83.5% versus 27.8%) [30]. Furthermore, the incorporation of chlorhexidine bathing significantly enhances the efficacy of mupirocin by addressing potential extranasal colonization.

An important limitation of this study is the absence of post-decolonization follow-up nasal swabs, which prevents objective confirmation of bacterial eradication. Consequently, the true effectiveness of the decolonization protocol cannot be fully quantified. Moreover, patient adherence to the prescribed regimen was not directly monitored and may have influenced the real impact of the intervention. In fact, some studies have indicated that decolonization is not achieved in all treated cases. For instance, in the study by Baratz et al., 22% of patients remained colonized despite following the decolonization protocol that included mupirocin ointment and chlorhexidine showers [23]. Similarly, Trautmann et al. observed an eradication rate of 85%, although no subsequent reduction in the rate of SSIs or in the length of hospital stay was noted [30].

Povidone iodine has been investigated as a potential alternative to the combination of mupirocin and chlorhexidine for resistant strains; however, only in vitro and ex vivo studies have been conducted so far, which have shown potential benefits [31].

Despite the promising potential of mupirocin and chlorhexidine for decolonization, there is no consensus in the literature regarding their effectiveness in reducing PJIs and SSIs caused by *S. aureus*.

Many studies have reported no statistically significant reduction in infection rates. For instance, Mihara et al. conducted research involving 6251 patients and found no significant correlation between nasal MRSA contamination and PJIs. Among patients undergoing elective primary THA, only 106 tested positive for MRSA in nasal swabs. Of those, three showed resistance to mupirocin ointment, and only one out of 22 patients who developed a PJI had a positive swab result [32]. Similarly, Perl et al. did not find a significant reduction in SSI rates. They conducted a double-blind study involving 891 patients who tested positive in screening, with one group receiving mupirocin and the other receiving a placebo. In this study, a reduction in the infection rate of 3.7% was observed (*p* = 0.02) [33].

Conversely, Price et al. advocate for the eradication of MRSA prior to surgery, as they observed a reduction in the rate of SSIs caused by MRSA [25]. Sporer et al. reported an impressive 69% decrease in SSIs [34]. Additionally, Jeans et al. analyzed 12,910 primary arthroplasties and found that the pre-screening rate of PJI due to MSSA was 0.75%, which decreased to 0.25% following the implementation of screening (*p* < 0.0001). The overall rate of PJI also declined from 1.92% to 1.41% (*p* = 0.03) [35].

To reduce costs and improve the cost–benefit ratio of universal screening, Von Rehlingen-Prinz et al. opted to administer a questionnaire to 37,745 patients scheduled for arthroplasty surgery instead of conducting universal screening. Based on prior hospitalizations and a history of previous *S. aureus* infections, they assessed the risk of postoperative MRSA infection. Consequently, they selectively performed preoperative testing on only 21.3% of the patients, achieving a positivity rate of 0.81% and a cost reduction of 79% [36].

This study has several limitations. Firstly, we did not conduct a nasal swab after the decolonization treatment, which means we lack data on the treatment’s effectiveness in eradicating the bacteria. Additionally, the actual adherence of patients to the therapy is difficult to quantify. Furthermore, the lack of a clonal relationship between the nasal and infecting strains limits the ability to analyze the origin (endogenous or exogenous) of each S. aureus PJI. Also the follow-up duration may limit capturing the full spectrum of postoperative infections.

Future research should focus on large-scale multicenter prospective studies to better define the clinical and economic impact of routine Staphylococcus aureus screening in elective arthroplasty. Randomized controlled trials comparing universal screening, targeted screening, and risk-based questionnaires may help determine the most cost-effective strategy. Additionally, molecular typing of colonizing and infecting strains would clarify whether infections originate from endogenous reservoirs. Standardization of decolonization protocols and monitoring of mupirocin resistance patterns should also be incorporated into future investigations.

## Conclusion

The implementation of a structured preoperative nasal screening and decolonization protocol for Staphylococcus aureus was associated with the absence of related periprosthetic joint infections during the intervention year. Although statistical significance was not achieved, the observed trend suggests a potential clinical benefit. The prevalence of nasal carriage in this cohort was consistent with previously reported data. The overall infection rate remained low across the study period, reflecting adherence to standardized perioperative prevention measures. Further high-quality prospective multicenter studies are necessary to establish definitive recommendations and optimize infection prevention strategies in elective total joint arthroplasty.

## Data Availability

The datasets generated and/or analyzed during the current study are not publicly available due to institutional policies and patient privacy considerations, but are available from the corresponding author on reasonable request.
